# Genetic and Comparative Transcriptome Analysis Revealed DEGs Involved in the Purple Leaf Formation in *Brassica juncea*

**DOI:** 10.3389/fgene.2020.00322

**Published:** 2020-04-24

**Authors:** Shuangping Heng, Lei Wang, Xi Yang, Hao Huang, Guo Chen, Mengdi Cui, Mingfang Liu, Qing Lv, Zhengjie Wan, Jinxiong Shen, Tingdong Fu

**Affiliations:** ^1^College of Life Sciences, Institute for Conservation and Utilization of Agro-Bioresources in Dabie Mountains, Xinyang Normal University, Xinyang, China; ^2^College of Horticulture and Forestry, Huazhong Agricultural University, Key Laboratory of Horticultural Plant Biology, Ministry of Education, Wuhan, China; ^3^College of Plant Science and Technology, Huazhong Agricultural University, Wuhan, China

**Keywords:** anthocyanin, purple leaves, *Brassica juncea*, transcriptome, gene expression

## Abstract

*Brassica juncea* is an important dietary vegetable cultivated and consumed in China for its edible stalks and leaves. The purple leaf mustard, which is rich in anthocyanins, is eye-catching and delivers valuable nutrition. However, the molecular mechanism involved in anthocyanin biosynthesis has not been well studied in *B. juncea*. Here, histological and transcriptome analyses were used to characterize the purple leaf color and gene expression profiles. Free-hand section analysis showed that the anthocyanin was mainly accumulated in the adaxial epidermal leaf cells. The anthocyanin content in the purple leaves was significantly higher than that in the green leaves. To investigate the critical genes and pathways involved in anthocyanin biosynthesis and accumulation, the transcriptome analysis was used to identify the differentially expressed genes (DEGs) between the purple and green leaves from the backcrossed BC3 segregation population in *B. juncea*. A total of 2,286 different expressed genes were identified between the purple and green leaves. Among them, 1,593 DEGs were up-regulated and 693 DEGs were down-regulated. There were 213 differently expressed transcription factors among them. The MYB and bHLH transcription factors, which may regulate anthocyanin biosynthesis, were up-regulated in the purple leaves. Interestingly, most of the genes involved in plant–pathogen interaction pathway were also up-regulated in the purple leaves. The late biosynthetic genes involved in anthocyanin biosynthesis were highly up-regulated in the purple leaves of *B. juncea*. The up regulation of *BjTT8* and *BjMYC2* and anthocyanin biosynthetic genes (*BjC4H*, *BjDFR*, and *BjANS*) may activate the purple leaf formation in *B. juncea*. This study may help to understand the transcriptional regulation of anthocyanin biosynthesis in *B. juncea*.

## Introduction

Anthocyanin, which includes numbers of natural water-soluble pigments, colored the fruit and flowers of many plants (Glover and Martin, 2012). Anthocyanin could produce various colors, such as red, purple, blue, yellow, and orange, in different plant organs and is beneficial to health (Castañeda-Ovando et al., 2009). High-anthocyanin food consumption could help to stimulate the immune system and reduce cancer risks (Thomasset et al., 2009). Dietary anthocyanins, which are important health-promoting antioxidants, make major contribution to the quality of fruits or leaves. Furthermore, dietary anthocyanin accumulation is also recognized as a visible biomarker of plants that have suffered from environmental stresses in many studies (Xie Y. et al., 2016; Zheng et al., 2019; Sun et al., 2020). Anthocyanin biosynthesis and regulation pathway has been well studied in *Cruciferae* plants including *Arabidopsis*, *Brassica rapa*, *Brassica oleracea*, *Brassica napus*, etc. (Guo et al., 2014; Xie Y. et al., 2016; Goswami et al., 2018; Yan et al., 2019).

In *Arabidopsis*, anthocyanin biosynthesis genes can be divided into early biosynthesis genes (EBGs) and late biosynthesis genes (LBGs). The ternary complex of MYB-bHLH-WD40 influences anthocyanin biosynthesis pathway through regulating the expression intensity and pattern of the structural genes by binding to their promoter regions (Petroni and Tonelli, 2011). Anthocyanin accumulation is also induced by different environmental stress (Xie Y. et al., 2016). In *Arabidopsis*, overexpression of the R2R3-MYB transcription factors (*PAP1*, *PAP2*, *MYB113*, and *MYB114*) increased anthocyanin accumulation (Gonzalez et al., 2008). Overexpression of *MYB75* increased anthocyanin and flavonol levels which enhance plant resistance to a specialist caterpillar (*Pieris brassicae*) in *Arabidopsis* (Onkokesung et al., 2014). The up-regulation of *BoMYB2* and *BoTT8* leads to the anthocyanin accumulation in red cabbage and purple cauliflower (Yuan et al., 2009; Chiu et al., 2010). Transcriptional regulation of *BrTT8* promotes anthocyanin biosynthesis in purple bok choy (*B. rapa* var. *chinensis*) (Zhang et al., 2014). And the transcript level of *BjTT8* is associated with up-regulation of most anthocyanin biosynthetic genes (Xie et al., 2014). The loss expression of *BoMYBL2-1* gene resulted in the establishment of purple *B. oleracea* (Song et al., 2018). In *Arabidopsis*, the *TT8* is required for expression of flavonoid late biosynthetic genes, *DFR* and *BAN* (Nesi et al., 2000). The expression profiling of genes involved in transcriptional regulation and structure genes of anthocyanin biosynthesis were identified in the high-anthocyanin resynthesized *B. napus* (Goswami et al., 2018). It was reported that *BrEGL3.1* and *BrEGL3.2*, which encode bHLH transcription factors, are two candidate genes for anthocyanin accumulation in zicaitai (Guo et al., 2015). These studies provide insight into the mechanisms of coloration in *Cruciferae* crops.

In recent years, RNA-seq has been used to identify differentially expressed genes (DEGs) involved in anthocyanin biosynthesis. The metabolism combined with transcriptome methods had been used to identify novel genes involved in flavonoid biosynthesis in *Arabidopsis* (Tohge et al., 2005). Transcriptome analysis showed that most of the anthocyanin biosynthetic genes were highly up-regulated in purple leaves investigated in *Brassica* (Mushtaq et al., 2016). Transcriptome analysis has been used for comprehensive understanding of the molecular mechanisms of stem development in *B. juncea* (Sun et al., 2012). Transcriptome had also been used to identify genes for the biosynthesis of flavonoids of *B. juncea* seed coat (Liu et al., 2013). Transcriptomic and metabolomic study revealed that the main genes involved in flavonoid biosynthesis were up-regulated in red kale than green kale (Jeon et al., 2018a). RNA-seq analysis was employed to seek the candidate genes involved in dark-purple leaves from *B. rapa* (Xie L. et al., 2016). Mustard (*B. juncea*) is widely cultivated as a typical condiment, cruciferous vegetables, and oilseed crops worldwide (Chen et al., 2013). The cultivated mustard varieties have been widely used as a fresh and pickled vegetable in the southwest of China (Fan et al., 2017). The water-soluble anthocyanins are widely distributed in the purple leaf mustard. The anthocyanin-rich mustard is beneficial to heath due to its high antioxidant activity and colorful germplasm resource for health vegetables breeding. A previous study has identified 67 anthocyanins, 102 flavonol glycosides, and 40 hydroxycinnamic acid derivatives from the red mustard greens (*B. juncea* Coss variety) (Lin et al., 2011). And more than 20 different types of anthocyanins were separated and identified from the purple tumorous stem mustard by high performance liquid chromatography electrospray ionization tandem mass spectrometry (HPLC-ESI-MS/MS) (Xie et al., 2014). The genome sequence of *B. juncea* has facilitated us to select anthocyanin biosynthesis genes in it (Yang et al., 2016). Although a number of genes involved in anthocyanin biosynthesis and regulation pathway have been studied in *Brassica* species, only few study mining candidate genes involved in anthocyanin formation in mustard by using RNA sequencing. Thus, comprehensive understanding of anthocyanin biosynthesis and regulation will promote the utilization of the anthocyanin in mustard. To better understand the molecular mechanism of anthocyanin biosynthesis, RNA-seq technology was employed to analyze the expression profile of different expressed genes between purple leaves and green leaves. These results may facilitate to promote molecular function analysis of the key genes involved in biosynthesis of anthocyanin and elucidate the molecular mechanism about it in *B. juncea*.

## Materials and Methods

### Plant Materials

The purple leaf (ZiYi) line and the green leaf (LvYi) line were planted in the experimental field at Xinyang Normal University (Xinyang, China). The ZiYi and LvYi plants were crossed to produce the F1 generation. The F1 plants were backcrossed with the recessive LvYi plants to construct the BC1 population in 2016. Then, the purple plants from the BC2 population planted in 2017 were backcrossed with the recessive LvYi parent line to construct the BC3 population in year 2018 (Supplementary Figure S1). The leaves from the purple and green plants in the BC3 population were frozen in liquid nitrogen and stored at −80°C until use for RNA extraction in Dec 2018.

### Extraction and Measurement of Anthocyanin Content

A total of anthocyanins was extracted from the purple and green plants in the BC3 population with three replicates, respectively. The purple and green leaves from the BC3 population were extracted into a mixed liquid with 1 ml of 95% ethanol and 1.5 M HCl (4:1, v/v) for 24 h at 4°C in the dark. Then, the extracts were centrifuged at 12,000 g for 10 min at 4°C. The absorption of the supernatant extracts at 530 nm was determined using a Thermo Scientific^TM^ NanoDrop^TM^ One. Total anthocyanin content was measured by the methods described previously (Li et al., 2016). The data were analyzed by independent samples *T* test and significant differences with *P* < 0.01. Error bars indicated the standard error of the average anthocyanin contents.

### RNA Extraction and RNA-Seq

Total RNA was extracted from the ZiYi and LvYi plants in the BC3 population with three replications each. TaKaRa MiniBEST Plant RNA Extraction Kit (Code No: 9769) was used to extract total RNA following the manufacturer’s protocols. Agilent 2100 RNA Nano 6000 Assay Kit (Agilent Technologies, Santa Clara, CA, United States) was used to detect the integrity and concentration of RNA samples. RNA seq was performed by using the Illumina Hiseq X Ten platform.

### Bioinformatics Analysis of Differential Expression Genes

Raw data were processed with the adopted filtering criteria as follows: remove the adaptor-polluted reads, remove the low-quality reads, and remove reads with number of N bases accounting for more than 5%. The obtained clean data will be carried out on statistics to analyze its quantity and quality, including Q30, data quantity and base content statistics, etc. Bowtie/Bowtie2 was used for building the genome index files. Clean Data was mapped to the *B. juncea* genome^1^ by using tophat v2.0.12. Fragments count for each gene in each sample was counted by HTSeq v0.6.0, and FPKM (fragments per kilobase per million mapped fragments) was calculated to estimate the expression level of genes in each sample. DESeq/DESeq2 was used for DEGs between ZiYi and LvYi with three biological replicates using a model based on the negative binomial distribution. The *P*-value could be assigned to each gene and adjusted by the Benjamini and Hochberg’s approach for controlling the false discovery rate. Genes with a fold change >2 and a *P*-value < 0.05 were identified as DEGs. GO^2^ (Gene Ontology) enrichment of DEGs was implemented by the hypergeometric test, in which *P*-value is calculated and adjusted as *q*-value, and data background is genes in the whole genome. GO terms with *q* < 0.05 were considered to be significantly enriched. GO enrichment analysis could exhibit the biological functions of the DEGs. KEGG^3^ (Kyoto Encyclopedia of Genes and Genomes) is a database resource containing a collection of manually drawn pathway maps representing our knowledge on the molecular interaction and reaction networks. The KEGG enrichment of DEGs was implemented by the hypergeometric test, in which *P*-value was adjusted by multiple comparisons as *q*-value. KEGG terms with *q* < 0.05 were considered to be significantly enriched.

### Quantitative Real-Time PCR

The cDNA from different samples were synthesized from total RNA (3 μg) by using a PrimeScript^TM^ 1st Strand cDNA Synthesis Kit (Code No: 6110A). Then, they were diluted to 80 times. Each qPCR included 8.4 μl of the diluted cDNA template, 0.8 μl of each primer (10 μM), and 10 μl of SYBR Green PCR master mix (ABI, United States). Reactions were carried out on the ABI PRISM 7300 Real-Time PCR System (Applied Biosystems). Quantitative real-time PCR (qRT-PCR) was performed as follows: 95°C for 5 min; 40 cycles at 95°C for 10 s, 60°C for 15 s, and 72°C for 30 s; and a final extension step at 72°C for 5 min. Results were analyzed using the CFX Manager software according to the 2^–Δ^ ^Δ^ ^*CT*^ method (Livak and Schmittgen, 2001). Three biological replicates were carried for gene expression analysis. The primers used for quantitative real time PCR (qRT-PCR) analysis were designed by Primer express software (Applied Biosystems, United States). The primers used in our study are listed in Supplementary Table S1. The *BjActin* gene was used as an internal control gene for mRNA expression (Heng et al., 2018).

## Results

### The Inheritance and Different Anthocyanin Content in the Purple Leaves in *B. juncea*

**FIGURE 1:**
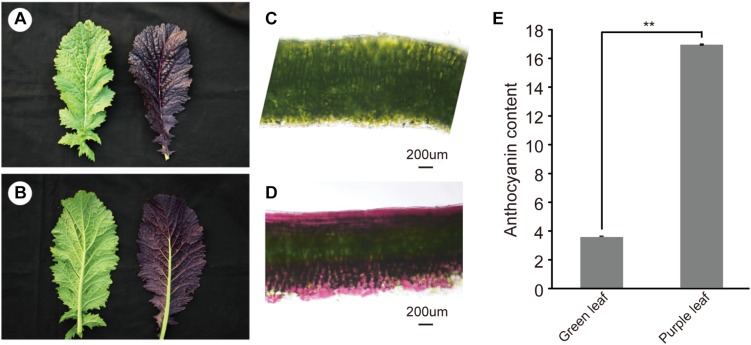
Morphology of purple leaf and anthocyanin content in *B. juncea*. **(A)** Upper epidermis of green (LvYi) and purple (ZiYi) leaf. **(B)** Lower epidermis of green and purple leaf. **(C)** Transverse section of a green leaf. Bars = 200 μm. **(D)** Transverse section of a purple leaf. Bars = 200 μm. **(E)** Total anthocyanin contents of green and purple leaves. **Signifcantly diferent at *P* < 0.01.

Purple leaf mustard showed higher accumulation of anthocyanin in leaves than that of the green leaf mustard (Figures 1A–D). With the leaf develops, the anthocyanin was mainly accumulated on both adaxial and abaxial epidermis and subepidermal cell layers (Figure 1D). Only in the middle vein (Figure 1B), the anthocyanin appeared to be not accumulated on the abaxial side. The plants from the segregation population were further used to investigate the genetic pattern of the purple leaf color. All the F1 plants showed purple leaf color. The F2 segregation population showed a normal Mendelian segregation ratio of 3:1 (purple vs. green leaves). The backcrossed segregation population showed a normal Mendelian segregation ratio of 1:1. Our result showed that a dominant gene controlled the purple leaf trait in *B. juncea* (Supplementary Table S2). To accurately determine the anthocyanin content in ZiYi, total anthocyanin was extracted from purple leaf and green leaf mustard. The total anthocyanin content from ZiYi was 4.4 times higher in purple leaves when compared to the green leaves in mustard (Figure 1E).

### *De novo* Sequencing, Assembly, and Identification of Differentially Expressed Genes

**FIGURE 2:**
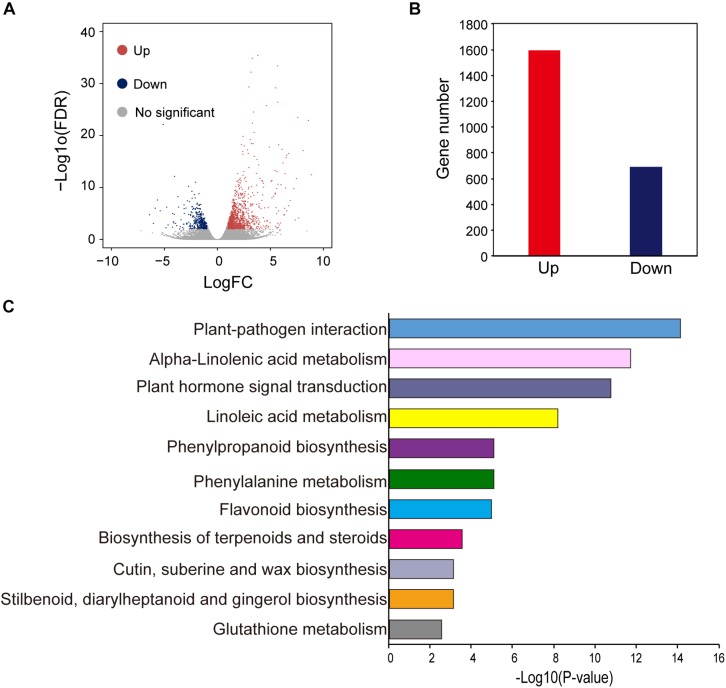
Different expressed genes between purple and green leaf. **(A)** The volcano map of DEGs between ZiYi and LvYi. Each dot represents a gene. **(B)** The number of up- and down-regulated genes in ZiYi. **(C)** KEGG metabolism pathway analysis of the DEGs between ZiYi and LvYi. Histograms represent the metabolism pathway distribution; the X axial represents the number of genes that have been annotated to the metabolism pathway.

To obtain a genome-wide gene expression profile for purple leaf color formation, six sequencing libraries were constructed from purple leaf and green leaf mustard with three replicates each. A total of 150.01 million and 136.57 million clean reads were obtained by Solexa/Illumina sequencing of samples. About 256.73 million clean reads were selected after the initial screening with clean read ratios more than 95.82% (Table 1). As a result, we obtained 1.91–5.24 × 10^6^ mapped reads for the six libraries. Then, these sequences were assembled into the *B. juncea* genome from *Brassica* database (BRAD). In total, 81–83% reads for each individual library could be aligned against the *B. juncea* genome. The gene expression levels of purple leaf and green leaf mustard were estimated by calculating the FPKM (fragments per kilobase per million mapped fragments) values (log2 Fold change ≥ 1 and FDR ≤ 0.01). A total of 2,286 DEGs (Figure 2A) were identified between the purple leaves and green leaves (Supplementary Table S3). Among them, 1,593 genes were found to be up-regulated and 693 genes were significantly down-regulated in the purple leaves (Figure 2B).

**TABLE 1:** Summary of RNA-seq data from purple and green leaves in *B. juncea.*

**Samples**	**Clean reads**	**Clean bases**	**>Q30 (%)**	**Mapped read**	**Mapping rate (%)**
BjG1	40,875,732	6,131,359,800	96.14	33,976,472	83
BjG2	39,574,546	5,936,181,900	95.99	32,916,737	83
BjG3	42,524,054	6,378,608,100	96.02	35,463,035	83
BjP1	43,795,288	6,569,293,200	95.95	35,558,830	81
BjP2	44,779,946	6,716,991,900	96.16	37,022,946	83
BjP3	45,186,838	6,778,025,700	95.82	37,567,008	83

### Different Biological Process and Pathways Between Purple and Green Leaves

Gene ontology terms were used to analyze the functional classification of the DEGs in purple and green leaves, respectively. GO analysis divided these DEGs based on biological process (BP), cellular component (CC), and molecular function (MF) (Supplementary Figure S2). The largest BP subcategory for the DEGs in BjP vs. BjG was involved in cellular processes, metabolic process, single-organism process, and response to stimulus. The largest CC subcategory for the DEGs in BjP vs. BjG was related to cell part, organelle, and membrane. And the largest MF subcategory for the DEGs in BjP vs. BjG was binding, catalytic, and nucleic acid binding transcription factor. The number of up-regulated genes involved in different processes of BP, CC, and MF were much higher than the number of down-regulated genes. These results suggested that the purple leaf may undergo more complex metabolic activities involved in anthocyanin biosynthesis.

To further explore the biological pathways involved in anthocyanin biosynthesis, DEGs were annotated in the Kyoto Encyclopedia of Genes and Genomes (KEGG) by using KeggArray software. Among them, the plant-pathogen interaction (62 members), alpha-linolenic acid metabolism (22 members), plant hormone signal transduction (72 members), and linoleic acid metabolism (12 members) were the most significant changed typical pathways between the purple and green leaves (Figure 2C). In particular, pathways enriched in phenylpropanoid biosynthesis (25 members), phenylalanine metabolism (30 members), and flavonoid biosynthesis (12 members), which are hallmarks of metabolic plasticity that provide plant color and adapt to biotic and abiotic stress and promote health benefits, were significantly affected during purple leaf color formation. Most of these DEGs involved in the typical pathways mentioned above were up-regulated (Figure 2C).

### The Expression Profiles of Different Transcription Factor Families

**FIGURE 3:**
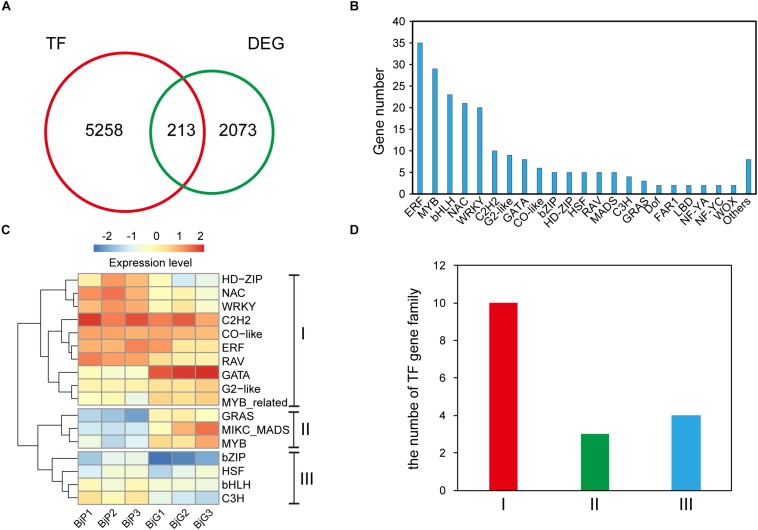
Distinct expression patterns of transcription factors. **(A)** The number of different expressed transcription factors between ZiYi and LvYi. **(B)** Gene numbers of different type transcription factors among the DEGs between ZiYi and LvYi. **(C)** Heatmaps representing the distinct expression patterns of transcription factors between ZiYi and LvYi. BjP1, BjP2, and BjP3 representative three biological replication in ZiYi. BjG1, BjG2, and BjG3 representative three biological replication in LvYi. **(D)** Comparison of the clusters of TF families.

To better understand the gene regulatory networks involved in anthocyanin biosynthesis, the different expressed transcription factors were further identified. Blast analysis the genome sequence of *B. juncea* revealed that there were 5,258 transcription factors in it. And a total of 213 differentially expressed transcription factors (Supplementary Table S4) was also found between the purple leaves and green leaves (Figure 3A). They were divided into more than 22 different types (Figure 3B). The ERF, MYB, and bHLH transcription factors are the three most TFs among them. Among the 35 differentially expressed ERF transcription factors, more than 60% of them were up-regulated in the purple leaf mustard. Cluster analysis found that these TF families could be classified into three clusters with distinctive expression patterns (Figure 3C). The number of each TF family members showed substantial diversity (Figure 3D). Blast analysis suggested that these ERF transcription factors were mainly involved in abiotic stress. It is known that numbers of MYB and bHLH transcription factors had been reported in anthocyanin biosynthesis (Albert et al., 2014). These differently expressed TFs may be involved in regulating anthocyanin biosynthesis genes in *B. juncea*. And *BjuB004115* encoding a bHLH regulation factor, which is homologous to *AtTT8* that regulate proanthocyanidin and anthocyanin biosynthesis (Nesi et al., 2000), is up-regulated in purple leaf mustard. *BjMYC2* (BjuO001663), which encodes a basic helix-loop-helix (bHLH) DNA-binding family protein (MYC2), was also up-regulated in purple leaf mustard. The upregulation of *BjTT8* and *BjMYC2* may contribute to the anthocyanin accumulation in purple leaf.

### Analysis of Differentially Expressed Enzymes Involved in Anthocyanin Biosynthesis

**FIGURE 4:**
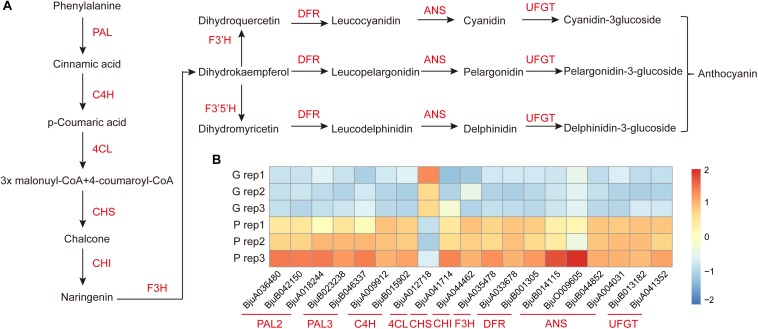
Diagram and heatmaps of genes involved the anthocyanin biosynthesis pathway. **(A)** Schematic diagram of genes involved in the anthocyanin biosynthesis pathway. **(B)** Heatmaps of DEGs involved in the accumulation of anthocyanin in ZiYi *B. juncea*.

To further investigate the key genes involved in anthocyanin biosynthesis and their regulation pathway, the genes involved in anthocyanin biosynthesis in *B. juncea* were blasted against their orthologous genes from *Arabidopsis* (Supplementary Table S5). The DEGs involved in anthocyanin biosynthesis were screened from 2,286 DEGs between purple and green mustard. The anthocyanin is biosynthesized through the flavonoid pathway. The key enzymes such as phenylalanine ammonia-lyase (*PAL*), cinnamate 4-hydroxylase (*C4H*), and 4-coumaryol CoA ligase (*4CL*) were involved in the phenylpropanoid pathway. And anthocyanin synthesis, which includes chalcone synthase (*CHS*), chalcone isomerase (*CHI*), flavanone 3-hydroxylase (*F3H*), and flavanone 3′-hydroxylase (*F3*′*H*), was involved in the early anthocyanin biosynthesis pathway. And dihydroflavonol 4-reductase (*DFR*), anthocyanidin synthase (*ANS*), and UDP-glucose flavonoid 3-O-glucosyl transferase (*UFGT*) were involved in the late anthocyanin biosynthesis pathway (Figure 4A). In our study, the transcript expression levels of most of the main enzymes involved in anthocyanins biosynthesis were up-regulated in purple leaves when compared with green leaves (Figure 4B). Among them, at least one copy of each key enzyme was up-regulated in purple leaves. It was found that three of the *PAL* genes were up-regulated in purple leaves. And four of the *ANS* genes were up-regulated in purple leaves. These up-regulated enzymes may promote the anthocyanins biosynthesis in purple leaf mustard. Apart from them, the *4CL* was found to be down-regulated in purple leaves. The *F3*′*H*, which encodes flavanone 3′-hydroxylase, was not up-regulated in purple leaves significantly. And the *F3*′*5* ′*H* gene, which encodes flavanone 3′, 5′-hydroxylase, was not found in our transcriptome data. These results suggested that the pelargonidin-3-glucoside biosynthesis pathway is the main pathway involved in anthocyanin biosynthesis, which contributes to purple leaf color formation in *B. juncea*.

### qRT-PCR Analysis of DEGs Involved in Anthocyanin Biosynthesis

**FIGURE 5:**
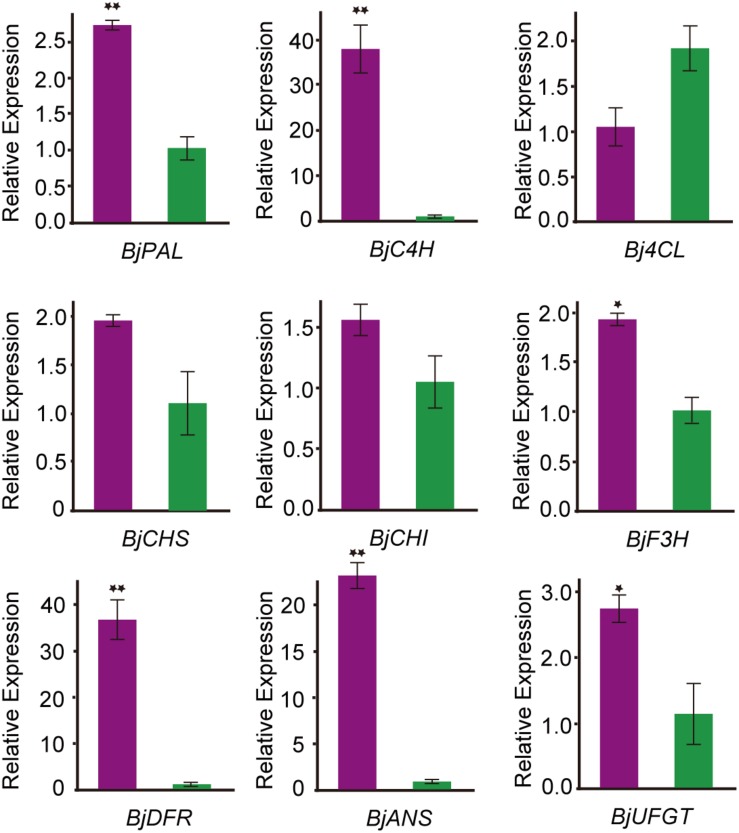
qRT-PCR analysis the expression pattern of genes involved in anthocyanin biosynthesis *B. juncea*. The purple color represents transcript expression level of genes in ZiYi *B. juncea* with purple leaf. The green color represents transcript expression level of genes in LvYi *B. juncea* with green leaf. *Signifcantly diferent at *P* < 0.05; **Signifcantly diferent at *P* < 0.01.

To confirm the transcript expression patterns of DEGs involved in anthocyanin biosynthesis, the key genes involved in anthocyanin biosynthesis-related enzymes were further analyzed through qRT-PCR. Nine DEGs related to anthocyanin biosynthesis pathways were selected for qRT-PCR analysis. The expression pattern of all these DEGs was consistent with the transcriptome data (Figure 5). Among them, phenylalanine ammonia-lyase (*PAL*), cinnamate 4-hydroxylase (*C4H*), dihydroflavonol 4-reductase (*DFR*), and anthocyanidin synthase (*ANS*) were significantly higher expressed in the purple leaf. But the 4-coumaryol CoA ligase (*4CL*) gene, which was involved in the phenylpropanoid pathway, was down-regulated in purple leaf. These results further confirmed the validity of our transcriptome data. And the high transcription level of *BjC4H*, *BjDFR*, and *BjANS* may be a direct cause of the purple leaf formation in *B. juncea*.

## Discussion

It has been known that anthocyanins could promote human health with high antioxidant activities (Khoo et al., 2017). Thus, studying the molecular mechanism of the anthocyanin biosynthesis and cultivating anthocyanin-rich plants is necessary for healthy food production. Transcriptome sequencing has been used as a powerful tool to study genome-wide gene activities and helps to understand the molecular mechanisms underlying different biological process. However, knowledge of the transcriptome analysis of purple leaf formation in *B. juncea* is still lacking. Since the anthocyanin contents of purple leaf *B. juncea* greatly increased, this study was initiated with the aim to explore differently expressed anthocyanin regulate and biosynthesis genes in purple leaf *B. juncea*.

Many structural and regulatory genes participating in anthocyanin biosynthesis have been described in *Arabidopsis* and other *Brassica*. It has been reported that there was a single dominant gene control purple leaf color inheritance in *B. juncea*, Chinese Ziye mustard (Luo et al., 2011). A spontaneous mutant 1280-1 with purple leaves was controlled by a dominant gene (*BjPl1*), and it was mapped to a 225-kb interval on linkage group B2 of *B. juncea* (Zhao et al., 2017). The phenotype of purple leaf in *B. juncea* “HunanQianyang” was also controlled by a single dominant gene inheritance and an R2R3-MYB transcription factor enhanced anthocyanin over accumulation in the *B. rapa* introgression line within the darker-purple (Xie L. et al., 2016). Genetic study indicated that the purple leaf *B. juncea* in our study was also controlled by a single dominant gene. The genetic pattern suggested that these purple leaf inbred lines may be controlled by the same causal gene. However, the purple leaves in *B. napus* were controlled by a candidate incomplete dominant gene *BnAPR2*, which encodes adenosine 5′-phosphosulfate reductase, localized at the end of chromosome A03 (Li et al., 2016). And the leaf color is apparently different from those reported in *B. juncea*. The different models of genetic inheritance suggested that the purple leaf gene involved in the molecular mechanism of the anthocyanin biosynthesis may be different.

It has been widely known that the anthocyanin biosynthesis is mainly controlled by a ternary complex of MYB-bHLH-WD40. In different plant tissues, the bHLH transcription factors could bind DNA either alone or interact with MYB transcription factors to regulate anthocyanin and PA biosynthesis (Heim et al., 2003). Up-regulated the regulatory gene *BjTT8* and all biosynthetic genes may contribute to accumulation of anthocyanin in the purple Tumorous Stem Mustard (Xie et al., 2014). Transcriptome analysis showed that *TT8* was highly up-regulated in all purple leaves from *B. rapa*, *B. napus*, *B. juncea*, *B. oleracea*, and *B. carinata* (Mushtaq et al., 2016). The *BjTT8* was also up-regulated in purple leaf of *B. juncea*. In our study, another bHLH factor *BjMYC2* was also up-regulated in the purple leaves. In *Citrus sinensis*, the highest expression of *CsMYC2* was correlated with the purple leaf formation in the “Poros” lemon (Cultrone et al., 2009). The upregulation of *BjTT8* and *BjMYC2* may contribute to the anthocyanin accumulation in purple leaf of *B. juncea*. The ERF TFs have also been reported to be important in response to abiotic stresses, such as high salt, drought, hypoxia, and low temperature, etc. The anthocyanins are involved in plant defensive mechanisms against abiotic and biotic stresses (Asem et al., 2015). Most of the ERF TFs up-regulated in the purple leaves may help to defend against environmental stress.

In addition, based on RNA-seq data sets related to the purple leaf formation, numbers of genes involved in phenylpropanoid biosynthesis, phenylalanine metabolism, and flavonoid biosynthesis were found to be different expressed between purple and green leaves. The DEGs revealed that most of the anthocyanin biosynthesis genes were up-regulated in purple leaves. In purple-fleshed sweet potato, a root-preferential expressed gene, which encodes dihydrokaempferol reductase (*IbDHKR*), contributes to the anthocyanin biosynthesis in tuber roots (Liu et al., 2017). The MBW complex could activate the transcription of late anthocyanin biosynthesis pathway genes including DFR, ANS, etc. (Patra et al., 2013; Xu et al., 2015). In our research, *BjDFR* and *BjANS* were significantly higher expressed in purple leaves. This results well explained that these up-regulated anthocyanin biosynthesis genes in our study synergistically affect the purple leaf color. But the 4-coumaryol CoA ligase (*4CL*) gene, which catalyzes p-Coumaric acid to form 4-coumaroyl-CoA, was down-regulated in purple leaf. However, the transcript expression level of *Br4CL1* from purple pakchoi cultivar “8389” was up-regulated (Jeon et al., 2018b). It was reported that *4CL5*, which participated in phenylalanine metabolism, was expressed at very low level in different *Brassica* species. And apart from *B. oleracea*, almost no difference in transcript expression level of *4CL5* was found between purple and green leaves (Mushtaq et al., 2016). The *4CL* gene played an important role in the pathways of lignin biosynthesis (Jiang et al., 2019). The down-regulated *4CL* in our study may decrease the lignin biosynthesis and increase anthocyanin biosynthesis. Surprisingly, the most significant typical pathways between the purple and green leaves were the plant-pathogen interaction, alpha-linolenic acid metabolism, plant hormone signal transduction, and linoleic acid metabolism. Up expressed genes involved in typical pathways may be helpful to the adaptability to various environmental stresses. In summary, these data indicated the different response mechanism occurred between the purple leaves and green leaves. A better understanding of the anthocyanin biosynthesis and regulate network in *Brassica* is a prerequisite to develop anthocyanin-rich vegetables.

## Conclusion

In conclusion, we identified different anthocyanin contents and transcriptome profile between purple and green leaves in *B. juncea*. Histological analysis showed that different leaf color was mainly caused by the accumulation of anthocyanin on the surface of the leaf. The differences in the transcriptome profile of the BC3 population were consistent with the different leaf color observed. The upregulation of *BjTT8* and *BjMYC2* may activate the anthocyanin biosynthesis in purple leaf.

## Data Availability Statement

RNA seq data were deposited in the NCBI Sequence Read Archive (SRA) under accession number: PRJNA544908.

## Author Contributions

SH, ZW, JS, and TF contributed to the design of the research. SH, HH, XY, CG, MC, ML, and QL performed the sample collection, RNA isolation, and gene expression analysis. SH and LW performed the bioinformatics analysis. SH wrote the manuscript. All authors read and approved the final manuscript.

## Conflict of Interest

The authors declare that the research was conducted in the absence of any commercial or financial relationships that could be construed as a potential conflict of interest.

## Footnotes

1 http://brassicadb.org

2 http://geneontology.org/

3 http://www.kegg.jp/

## Supplementary Material

FIGURE S1 Technology roadmap of construction of the BC3 population.

FIGURE S2 Gene ontology (GO) classification of different expressed genes between the purple and green leaves. The *x*-axis represents the enrichment of DEGs in each category, and the *y*-axis represents the enrichment ratio and numbers of each GO term. The jacinth bars represent the percentages of up-regulated genes in each category/the total number of up-regulated genes in each GO term. The light green bars represent the percentages of down-regulated genes in each category/the total number of down-regulated genes in each GO term.

TABLE S1 The primers used for qPCR in this study.

TABLE S2 Genetic analyses of the purple leaves in F2 and backcrossed segregating populations.

TABLE S3 Detail of DEGs identified between the purple leaves and green leaves.

TABLE S4 Differentially expressed transcription factors between the purple leaves and green leaves.

TABLE S5 Orthologous genes involved in anthocyanin biosynthesis in *B. juncea*.
