# Supplementary figures and images for: Genetic and Comparative Transcriptome Analysis Revealed DEGs Involved in the Purple Leaf Formation in Brassica juncea
Source: Front Genet. 2020 Apr 24;11:322. doi: 10.3389/fgene.2020.00322 (PMC7193680; doi:10.3389/fgene.2020.00322)

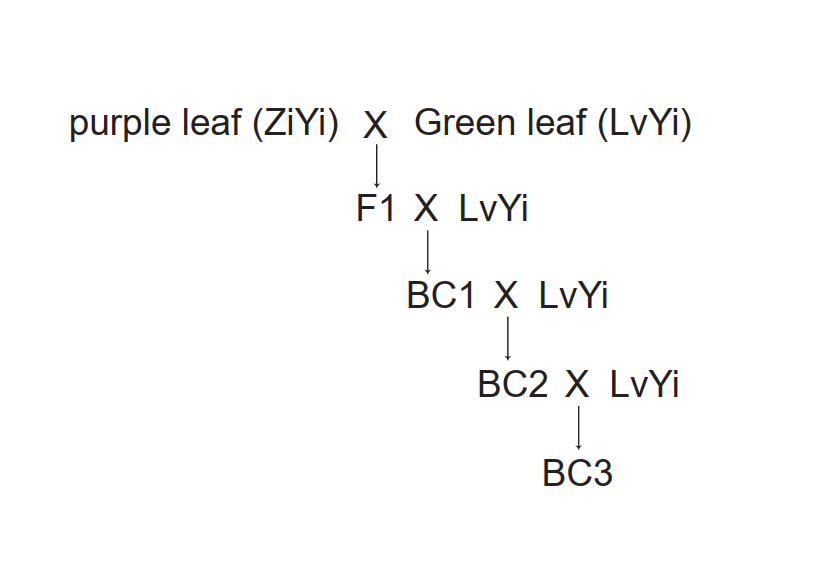

Supplement: FIGURE S1 — Technology roadmap of construction of the BC3 population. [file Image_1.TIF]

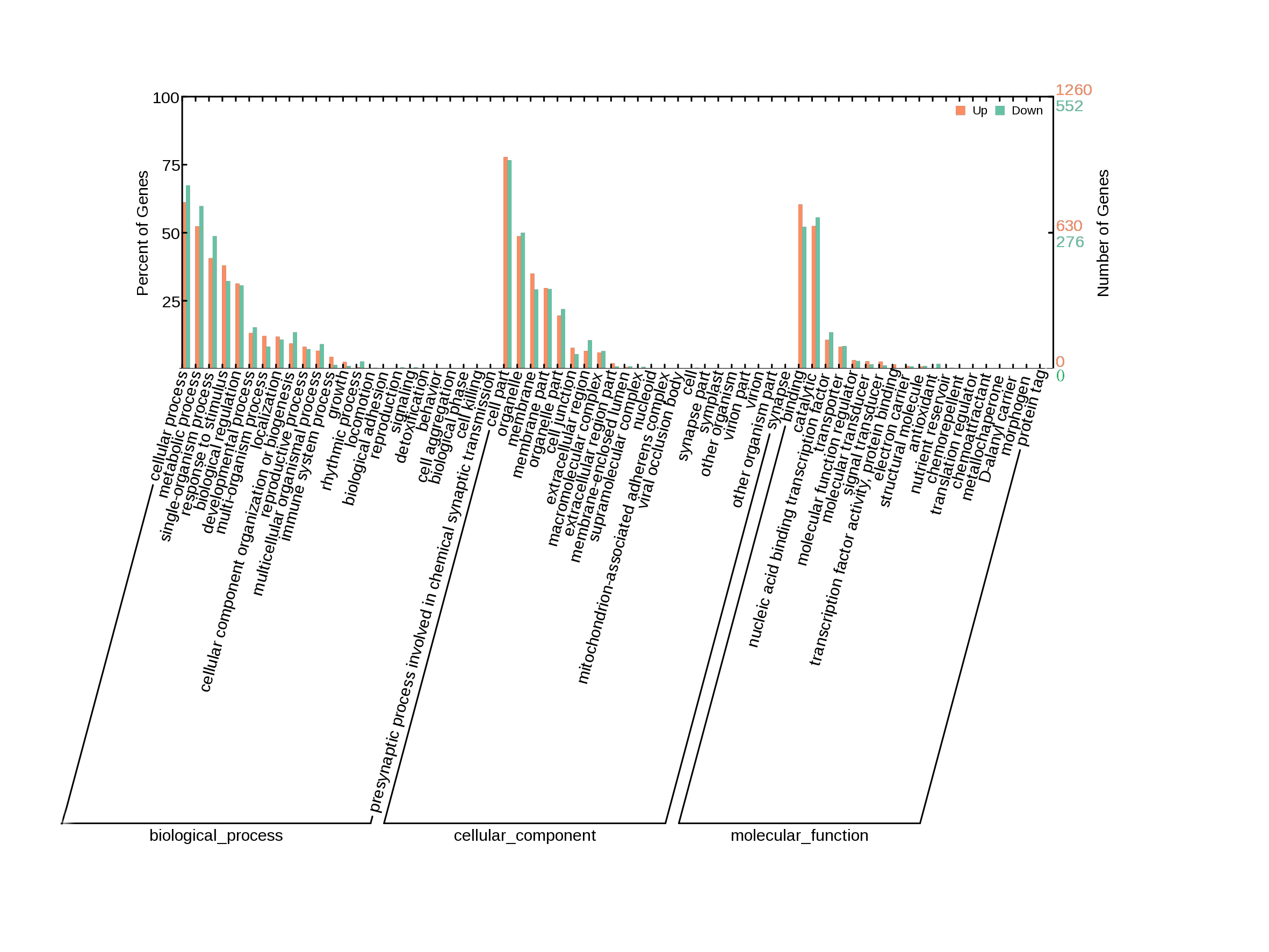

Supplement: FIGURE S2 — Gene ontology (GO) classification of different expressed genes between the purple and green leaves. The x-axis represents the enrichment of DEGs in each category, and the y-axis represents the enrichment ratio and numbers of each GO term. The jacinth bars represent the percentages of up-regulated genes in each category/the total number of up-regulated genes in each GO term. The light green bars represent the percentages of down-regulated genes in each category/the total number of down-regulated genes in each GO term. [file Image_2.TIF]
