# Supplementary material for: Genetic and Comparative Transcriptome Analysis Revealed DEGs Involved in the Purple Leaf Formation in Brassica juncea
Source: Front Genet. 2020 Apr 24;11:322. doi: 10.3389/fgene.2020.00322 (PMC7193680; doi:10.3389/fgene.2020.00322)
Supplement: TABLE S2 — Genetic analyses of the purple leaves in F2 and backcrossed segregating populations. [file Table_2.docx]

| Years | Population | segregation | | | Mendelian expectations | *χ*^2^  value |
| --- | --- | --- | --- | --- | --- | --- |
|  |  | Plants tested | Purple  ZiYi | Green  LvYi |  |  |
| 2015 | P_1_ | 10 | 10 | 0 | **—** | **—** |
|  | P_2_ | 10 | 0 | 10 | **—** | **—** |
|  | F_1_ | 50 | 50 | 0 | **—** | **—** |
| 2016 | F2 | 621 | 479 | 142 | 3:1 | 1.51 |
|  | BC1 | 124 | 55 | 69 | 1:1 | 1.58 |
| 2017 | BC2 | 206 | 112 | 94 | 1:1 | 1.57 |
| 2018 | BC3 | 184 | 97 | 87 | 1:1 | 0.54 |

χ^2^ <χ^0.05^ (3.84) is considered as significant
