# Supplementary material for: Genetic and Comparative Transcriptome Analysis Revealed DEGs Involved in the Purple Leaf Formation in Brassica juncea
Source: Front Genet. 2020 Apr 24;11:322. doi: 10.3389/fgene.2020.00322 (PMC7193680; doi:10.3389/fgene.2020.00322)
Supplement: TABLE S5 — Orthologous genes involved in anthocyanin biosynthesis in B. juncea. [file Table_5.docx]

Table S3 Orthlogous genes involved in anthocyanin biosynthesis in *B. juncea.*

| Gene Name | At Gene | Bj Gene |
| --- | --- | --- |
| PAL1 | AT2G37040 | BjuA015849 |
|  |  | BjuO006770 |
| PAL2 | AT3G53260 | BjuA036480 |
|  |  | BjuB042150 |
|  |  | BjuA018244 |
| PAL3 | AT5G04230 | BjuB023238 |
|  |  | BjuB046337 |
| PAL4 | AT3G10340 | BjuB016349 |
| C4H | AT2G30490 | BjuA018788 |
|  |  | BjuB015902 |
|  |  | BjuA009912 |
|  |  | BjuO013195 |
|  |  | BjuA034341 |
|  |  | BjuB026422 |
|  |  | BjuA009911 |
|  |  | BjuB015901 |
|  |  | BjuA034340 |
|  |  | BjuB026423 |
| 4CL1 | AT1G51680 | BjuO007573 |
|  |  | BjuO007572 |
| 4CL2 | AT3G21240 | BjuO007573 |
|  |  | BjuO007572 |
| 4CL3 | AT1G65060 | BjuA031882 |
|  |  | BjuB043976 |
| 4CL5 | AT3G21230 | BjuA012716 |
|  |  | BjuB008665 |
|  |  | BjuA012718 |
|  |  | BjuA027816 |
|  |  | BjuB026130 |
|  |  | BjuB026044 |
| CHS | AT5G13930 | BjuA041225 |
|  |  | BjuB042919 |
|  |  | BjuB012366 |
|  |  | BjuA047389 |
|  |  | BjuB014803 |
|  |  | BjuA041714 |
|  |  | BjuA039278 |
|  |  | BjuB012368 |
|  |  | BjuA008346 |
|  |  | BjuB018049 |
| CHI | AT3G55120 | BjuA004576 |
|  |  | BjuA044462 |
|  |  | BjuB045469 |
|  |  | BjuA044461 |
| F3H | AT3G51240 | BjuA035478 |
|  |  | BjuA032280 |
|  |  | BjuB048740 |
| DFR | AT5G42800 | BjuA033678 |
|  |  | BjuB001305 |
| ANS | AT4G22880 | BjuO009605 |
|  |  | BjuB044852 |
|  |  | BjuB014115 |
|  |  | BjuB014113 |
|  |  | BjuA004031 |
| UGT79B1 | AT5G54060 | BjuB041100 |
|  |  | BjuA009708 |
|  |  | BjuB041101 |
|  |  | BjuA009709 |
|  |  | BjuB041102 |
|  |  | BjuB041103 |
| UGT75C1 | AT4G14090 | BjuA028394 |
|  |  | BjuO011591 |
| UGT78D2 | AT5G17050 | BjuB013182 |
|  |  | BjuA041352 |
|  |  | BjuB013185 |

Note: At Gene represent the genes from *Arabidopsis*; Bj Gene represent the genes from *B. juncea*; The purple color indicate the gene was up regulated in the purple leaves of ZiYi; The green color indicate the gene was down regulated in the purple leaves of ZiYi.
